# Monitoring soil fauna with ecoacoustics

**DOI:** 10.1098/rspb.2024.1595

**Published:** 2024-09-04

**Authors:** Jake M. Robinson, Amy Annells, Timothy R. Cavagnaro, Craig Liddicoat, Heidi Rogers, Alex Taylor, Martin F. Breed

**Affiliations:** ^1^ College of Science and Engineering, Flinders University, Bedford Park, SA 5042, Australia; ^2^ The Aerobiome Innovation and Research Hub, College of Science and Engineering, Flinders University, Bedford Park, SA 5042, Australia

**Keywords:** ecoacoustics, bioacoustics, soil health, soil biodiversity, biomonitoring

## Abstract

Ecoacoustics—or acoustic ecology—aids in monitoring elusive and protected species in several ecological contexts. For example, passive acoustic monitoring (PAM), which involves autonomous acoustic sensors, is widely used to detect various taxonomic groups in terrestrial and aquatic ecosystems, from birds and bats to fish and cetaceans. Here, we illustrate the potential of ecoacoustics to monitor soil biodiversity (specifically fauna)—a crucial endeavour given that 59% of species live in soil yet 75% of soils are affected by degradation. We describe the sources of sound in the soil (e.g. biological, geological and anthropogenic) and the ability of acoustic technology to detect and differentiate between these sounds, highlighting opportunities and current gaps in knowledge. We also propose a roadmap for the future development of optimized hardware, analytical pipelines and experimental approaches. Soil ecoacoustics is an emerging field with considerable potential to improve soil biodiversity monitoring and ‘soil health’ diagnostics. Indeed, early studies suggest soil ecoacoustics can be successfully applied in various ecosystems (e.g. grasslands, temperate, tropical and arid forests) and land uses (e.g. agriculture, viticulture, natural and restored ecosystems). Given the low cost, minimal intrusiveness, and effectiveness in supporting soil biodiversity assessments and biosecurity risks, we advocate for the advancement of soil ecoacoustics for future land management applications.

## 1. Introduction

Approximately 95% of the Earth’s terrestrial habitats will be affected by degradation by 2050 [1]. In response to this global environmental crisis, the United Nations declared 2021–2030 the Decade on Ecosystem Restoration [2]. Soils are the foundation of terrestrial life. They are conglomerates of both abiotic (non-living) and biotic (living) entities that form dynamic ecosystems. An estimated 59% of planet Earth’s species live in soil [3] and invertebrates have major roles in soil health (e.g. via nutrient cycling, soil aggregation, plant health and food web dynamics) [4]. However, 75% of the world’s soils are already affected by degradation—a figure that could rise to 90% by 2050 if deforestation, overgrazing, urbanization and other harmful practices persist [5]. This poses a major problem for biodiversity and the ecosystem services that sustain human populations. Indeed, 98% of our calories come from soil [6], and earthworms alone underpin 6.5% of the world’s grain production [7]. However, detecting, measuring and monitoring soil biota is challenging—it’s costly and intrusive at scale using current methods [8,9]. Therefore, improved cost-effective and non-destructive soil biota assessments and tools to guide soil management are needed. Soil ecoacoustics can be considered both a toolkit and an emerging discipline within the broader field of ecoacoustics [10].

**Figure 1.**
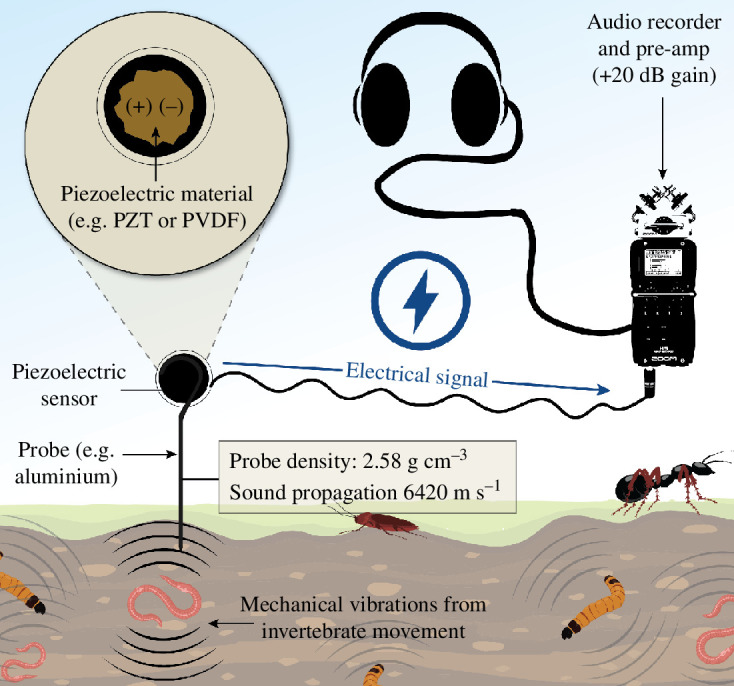
A basic soil ecoacoustics field recording set-up. This comprises an audio recorder with an impedance filter and pre-amp (e.g. +20 dB), a piezoelectric (contact) microphone, which typically contains a piezoelectric ceramic, such as lead zirconate titanate (PZT) or a polymer such as polyvinylidene fluoride (PVDF), a metal probe, ideally aluminium for its sound propagation properties, and some headphones. The mechanical vibrations caused by invertebrates are converted to an electrical signal and the data are stored on an secure digital (SD) card, ready for downstream analysis.

Ecoacoustics or passive acoustic monitoring (PAM) detects acoustic waves emitted by soniferous organisms (e.g. bats, cetaceans, birds, frogs and insects), and contributes to ecosystem assessments [11,12]. PAM is widely used to monitor above-ground and aquatic soundscapes, revealing changes in biodiversity [13] and ecosystem dynamics [14]. By detecting variations in species’ acoustic signatures, ecoacoustics provides valuable insights into the status and trends of individual species and communities [15]. Acoustic data can be analysed to infer the presence/absence and distribution patterns of species, and the diversity and composition patterns of ecological communities. There is potential to develop these approaches for soils (figure 1), where soil soundscapes typically include *biophony*, *geophony* and *anthrophony* (see Box 1 for details). Indeed, in healthy soils, biota are typically abundant and active, reflected in their acoustic profiles at the soundscape level [21].

Box 1.
**Sources of underground sounds.**

*Biophony* [16] is the sound generated by organisms within the soil. This includes the activities of invertebrates such as ants, beetles and earthworms (and potentially vertebrates, e.g. moles and mice), which produce acoustic signals while moving through and interacting with soil particles [17]. Some organisms also stridulate (i.e. through rubbing body parts together), which generates vibrations. Additionally, the growth and movement of plant roots generate subtle sounds as they push through soil and absorb nutrients and water [18]. These biological activities contribute to the acoustic environment of the soil, providing information about the health and biodiversity of soil ecosystems.

*Geophony* [18] encompasses the non-biological, natural sounds emanating from the earth itself. This includes the movement of soil particles, tremors caused by seismic activity, and the flow of water through soil and rock layers [19]. These geophysical sounds can vary from the subtle shifts of soil during erosion processes to the more pronounced rumblings of underground water flow or seismic events. Monitoring these sounds could help understand soil stability, hydrological processes, and seismic activity and enable more robust ecoacoustic data analysis (e.g. by separating geophony from biophony).

*Anthrophony* [20] includes all human-generated sounds that penetrate the soil environment. This can range from the mechanical sounds (‘technophonies’) produced by agricultural machinery, construction equipment and transportation networks to other sounds related to industrial activities [19]. These human-induced technophonies can significantly impact the acoustic landscape of the soil, often masking natural biophonic and geophonic sounds [17]. Understanding the extent and impact of anthrophony is crucial for assessing human influence on soil ecosystems and their acoustic properties and developing strategies to mitigate its negative effects.

Investigations into the potential of ecoacoustics to detect and analyse below-ground fauna are growing [17,22]. Indeed, soil ecoacoustics is a nascent but expanding field. A 2024 horizon scan of global biological conservation issues identified soil ecoacoustics as an emerging global priority [23]. Here, we synthesize the findings from soil ecoacoustics (and related) studies and show that ecoacoustics can contribute to soil biodiversity monitoring across various ecosystems and land uses. We also propose a roadmap for the future development of soil ecoacoustics hardware, analytical pipelines and experimental approaches.

### (a) Soil ecoacoustics for individual taxa

Soil ecoacoustics began by assessing acoustics methods to detect larval infestations in North American vineyards [24]. This pioneering work used accelerometer technology and microphones to detect sounds created by Coleopteran larvae *in situ*. They detected and profiled the sounds generated by the larvae, but the technique was limited by the effects of geophony and anthrophony. Other attempts to detect *Coleoptera* sp., acoustically, both *ex situ* and *in situ* supported the notion that ecoacoustics can monitor soil infestations [25–27]. However, these studies relied on sound profiles developed by the human auditory system without robust standardization. Görres and Chesmore [28] introduced an automated acoustic analysis for Scarabaeidae stridulations, utilizing fractal dimension-based analysis that focuses on the time domain of an audio recording independent of amplitude. However, these samples still underwent manual human auditory preprocessing for stridulation detection and count before applying automation. Ecoacoustics has successfully detected *Myrmica*, Annelids and Blattodea, highlighting the broad range of invertebrates detectable by the approach [29–31].

While these studies strongly support the potential of soil ecoacoustics, they highlight a clear need for research into robust and standardized analytical procedures. Machine learning algorithms for pattern detection have the potential to isolate biophony from background noise, removing the need for manual monitoring. Integration of machine learning in acoustic studies remains in its infancy, but it has proven effective in acoustic monitoring of woodpeckers and pollinating insects [32]. Furthermore, understanding the environment surrounding the invertebrates can potentially help differentiate geophony, anthrophony and invertebrate activity (e.g. by analysing factors such as habitat type, land use and weather conditions, researchers could more accurately attribute specific sounds to natural processes, human activities or invertebrate activity [33]).

In a study on tundra soils in Sweden, researchers used ecoacoustics to monitor the presence of invasive Arctic earthworms [34]. While soil soundscapes containing earthworms were shown to be more complex than those without, it was noted that earthworm activity was reduced during colder temperatures; thus, applications of acoustic monitoring may be limited in colder soil environments. Many studies of singular soil invertebrates involve detecting undesirable or ‘invasive’ species. However, despite the lack of literature on using soil ecoacoustics to monitor desirable species (e.g. those of conservation concern or with agricultural importance), the technology and concept are transferable to this objective. Moreover, there is potential to use ecoacoustics in behavioural sciences. Barbero *et al*. [29] explored this potential by investigating the predator–prey mimicry of the parasitic butterfly *Maculinea rebeli* and its host ant species *Myrmica schencki*. The study unveiled that the acoustic profiles of *M. rebeli* pupae and larvae were more similar to those of *M. schencki* queen ants than workers, allowing them to attain an elevated status within the colony community. Additional applications exploring ecoacoustics in this manner remain limited, however, as acoustic profiles are associated with behaviours (e.g. feeding, mating, commuting and presence/avoidance), soil ecoacoustics could be developed in ethological studies as a minimally intrusive method of assessing soil invertebrate behaviour.

## 2. Soil ecoacoustics in community ecology

### (a) Restoration and sustainable agriculture

Soil ecoacoustics has been applied to understand whole soundscapes. For instance, using acoustic indices, Maeder *et al*. [22] found that acoustic complexity was positively correlated with invertebrate abundance and diversity. Soil ecoacoustics has also been used in forest restoration contexts. A study in the United Kingdom compared soundscapes of deforested and restored sites both *in situ* and *ex situ* in a sound-attenuated chamber on-site, finding that acoustic complexity and diversity (as defined using the acoustic complexity index (ACI) and biodiversity index (BI), respectively) results were significantly higher in restored sites (an acoustic complexity score of 13 in deforested and 15 in restored sites) [17]. These results were supported by traditional invertebrate sampling methods, indicating higher invertebrate abundance in restored sites. Another study on a South Australian restoration project found that acoustic complexity and diversity were significantly higher in remnant and restoration sites than in degraded sites [35]. These results were observed both *in situ* and in sound chamber recordings. Ecoacoustic results in this study were positively correlated with invertebrate abundance and richness (*r* ^2^ = 0.60–69, *p* = 0.001), supporting ecoacoustics as an alternative approach to monitoring soil fauna.

A study in a tropical rainforest found that acoustic index scores significantly differed in tropical burnt versus unburnt forests [36]. The authors collected two soil acoustic datasets across three municipalities at long-term forest degradation monitoring sites. Sound recordings were analysed using a range of measures, including acoustic indices, the number of spectral events per second, frequency entropy and the proportion of spectrogram covered by regions of interest. Interestingly, acoustic diversity and complexity were both higher in burnt forests (acoustic diversity increased from 0.21 to 0.31, *p* = 0.02). The authors suggested that the increase in burnt forests might be due to the competitive release of generalist and/or fire-resilient species in these habitats following burns. This highlights the spatio-temporal complexities of soil ecoacoustics and the need for a deep understanding of context-dependent ecological dynamics.

Agroecosystems are home to complex assemblages of soil biota, many of which provide important ecosystem services [37]. For example, communities of earthworms, beetles, ants and other organisms play an important role in improving soil structure, organic matter decomposition and incorporation, and water infiltration [38,39]. Indeed, earthworms alone are lauded as key indicators of soil health in agricultural soils. Given their unique ecoacoustic signature, it may be possible to detect the impact of management practices that seek to improve soil health (e.g. through increased organism abundance and activity) using ecoacoustics. In contrast, agroecosystems are often challenged with invertebrate pests, with considerable effort and expense directed towards detecting and managing pest species. Ecoacoustics may provide another tool to detect and measure the activity of beneficial and/or pest invertebrates. For example, ecoacoustics could be used to assess the efficacy of pest eradication strategies through shifts in the soundscape pre- and postpest management intervention [40]. Agroecosystems probably have complex acoustic profiles, and day-to-day agricultural activities may ‘pollute’ the soundscape (i.e. anthrophony) [41]. However, with *in situ* sensing and instrumentation of agroecosystems increasingly common, especially for high-value intensive crops (e.g. grape vines), this provides an ideal opportunity for ecoacoustic sensors and approaches to be tested on a large scale.

To summarize, from an applied and functional ecology perspective, soil ecoacoustics can help identify the presence and abundance of species, providing insights into soil health and ecosystem functioning. Early detection of undesirable species that could disrupt local ecosystems is also possible [42]. Furthermore, sounds produced by soil organisms might reflect soil structure and other abiotic soil conditions (e.g. moisture content). For example, heavily compacted soils may have lower acoustic activity due to reduced habitat suitability for many organisms. Alternatively, we may detect stronger acoustic signals in denser soil due to increased sound propagation. In restoration, soil ecoacoustics has demonstrable potential for monitoring the return of soil biodiversity and activity levels as restoration progresses [17,33]. It can provide quantitative data on the effectiveness of restoration techniques by comparing acoustic profiles before and after interventions. A more speculative application is in climate change studies. For instance, soil ecoacoustics could conceivably help to record how temperature and moisture level changes influence soil biota activity. Moreover, soil ecoacoustics could track the activity of soil organisms involved in carbon cycling, contributing to studies on carbon sequestration and greenhouse gas emissions. However, the scientific evidence for these approaches is currently lacking, but these potential applications warrant further research.

### (b) Anthropogenic impacts on the acoustics of soil

**Figure 2.**
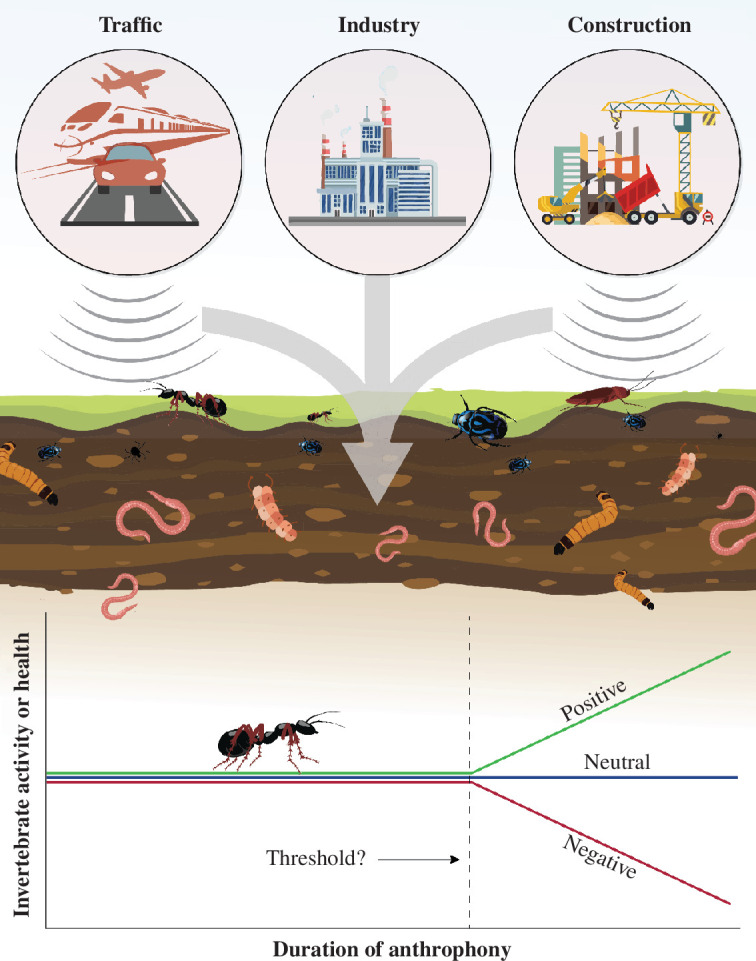
Sources of anthrophony/technophony that could potentially impact soil biota and ecological functions, with a graphical example of a research question relating to the duration of anthrophony on the activity or health of soil invertebrates and possible negative, neutral or positive outcomes.

Soil ecoacoustics has primarily focused on detecting biological sounds produced by soil-dwelling organisms. However, soil ecoacoustics also has untapped potential for detecting anthropogenic sounds or ‘anthrophony’ (‘technophonies’ when referring to the sound generated by mechanical or technological sources), which could negatively impact soil ecosystems [35]. Human activities such as construction, traffic and industrial operations, generate technophonies that can penetrate the soil environment [43] (figure 2). These technophonies could conceivably disturb soil biota, alter their behaviour and disrupt ecological processes. For instance, technophonies can alter ecological processes and wildlife activity, including movement, reproduction, offspring care and foraging [35,44]. Technophonies can also alter the growth rate, biomass and composition of microbial communities [45]. By extending the scope of soil ecoacoustics to include technophonies, researchers can comprehensively understand how human-induced sounds affect soil biodiversity and health. This expanded application could better characterize the ecological impacts of human activities, inform better land-use practices, guide mitigation strategies and help protect and restore the integrity of soil ecosystems. There are many unanswered research questions, such as: do technophonies have a negative, neutral or even positive impact on soil biota (figure 2)? Are there temporal, loudness or frequency thresholds that determine the impacts?

## 3. The future of soil ecoacoustics

### (a) Analytical tools

At the soundscape level, soil ecoacoustics researchers widely use acoustic indices. The ACI measures the variability and complexity of acoustic signals within a soundscape. Developed by Pieretti *et al*. [46], ACI focuses on frequency and amplitude variations of sounds over time. High ACI values indicate a diverse and complex soundscape, typically associated with more abundant and diverse species. It has historically been used to detect and monitor the presence of vocal species, such as birds [47]. The BI quantifies the diversity of biological sounds within an acoustic recording [48]. This index assesses the distribution and abundance of different soniferous species. High BI values correspond to a greater diversity of species.

The BI is advantageous for long-term monitoring and comparing biodiversity levels across different habitats or temporal scales. The acoustic diversity index (ADI) is designed to measure the diversity of sound sources within a given environment [49]. ADI calculates the number of frequency bands occupied by biological sounds in a soundscape. Higher ADI values indicate a greater diversity of species and a more complex acoustic environment. This index has been suggested to be useful for assessing biodiversity in various ecosystems and can be particularly effective in monitoring changes over time or in response to environmental disturbances [50]. The normalized difference soundscape index (NDSI) differentiates between biophonic (biological) and anthrophonic (human-made) sounds. Developed by Kasten *et al*. [51], the NDSI is calculated as the difference between the biophonic and anthrophonic sound energy, normalized by their sum. A high NDSI value indicates the dominance of biological sounds over human-generated technophonies. This index has been particularly useful for assessing the impact of anthropogenic technophonies on ecosystems [52] but is limited to situations where there is variable anthrophony.

**Figure 3.**
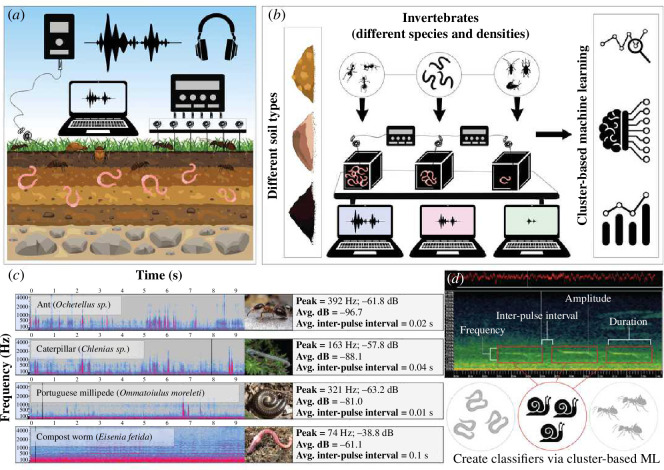
A myriad of methodological possibilities: (*a*) testing different sampling devices in the field, (*b*) running controlled mesocosm trials, (*c*) testing the acoustic signals of different taxa and studying their associated morphological and behavioural traits, and (*d*) creating classifiers using cluster-based machine learning techniques—based on acoustic parameters such as frequency, amplitude, inter-pulse interval and duration.

These indices were designed predominantly for above-ground biodiversity. Therefore, it would be prudent for future research to focus on developing updated metrics, indices and analytical tools specifically for soil environments. For example, emerging analytical ecoacoustic tools for differentiating between species include cluster-based classifiers (figure 3). Cluster-based machine learning for building ecoacoustic classifiers involves grouping similar acoustic data points into clusters to identify patterns and classify sounds within an ecosystem [53–55]. This method leverages unsupervised learning algorithms, such as k-means or hierarchical clustering, to partition the acoustic data into distinct clusters based on features like frequency, amplitude and temporal patterns (e.g. duration and inter-pulse interval). Once these clusters are formed, labelled training data can be used to assign meaning to each cluster, allowing the system to learn and recognize different biological and anthropogenic sounds. This approach enhances the ability of the classifier to distinguish between acoustic signatures. It is already being used in biodiversity monitoring in above-ground systems (e.g. differentiating between bird, bat and amphibian species) [56,57]. Cluster-based classifiers are particularly valuable in ecoacoustics for handling the vast and complex datasets typical in natural soundscapes. Such modelling approaches can be developed using readily available platforms, such as Python. However, others have developed software that includes cluster-based classifier-building capabilities with user-friendly interfaces, for instance, Wildlife Acoustics Kaleidoscope Pro (https://www.wildlifeacoustics.com) and unsupervised machine learning packages in R [58]. However, further developments for soil-based applications are needed, irrespective of the platform used.

Similar to other data-focused biological domains (e.g. genomics), soil ecoacoustics would benefit from developing an international open-access database of soil ecoacoustic data. This database could serve as a centralized repository where researchers globally could upload, share and access acoustic recordings from various soil environments. Such a resource would promote collaboration and standardization, enabling scientists to compare data across different regions and ecosystems effectively. Standardized protocols for data collection and quality control, plus the inclusion of comprehensive meta-data (e.g. location, land use and edaphic properties), would enhance the consistency and reliability of the shared data. Access to a comprehensive dataset would facilitate more extensive and comparative studies, to help identify patterns and trends that might elude smaller, isolated datasets.

### (b) Hardware

A range of audio recorders, sensors and probes have been used to detect soil biota in different environments [17,20]. Mankin *et al*. [24] used an accelerometer (a device that measures acceleration forces, enabling it to detect changes in velocity and orientation) to detect *Diaprepes abbreviatus* grubs in soil, and under laboratory or ideal field conditions, active insects within 30 cm were identified with nearly 100% reliability, falling to 75% in field tests under adverse conditions. Maeder *et al*. [22] used a piezo diaphragm from Murata (15 mm in diameter and 0.2 mm in thickness) along with a 10 cm long and 1 mm thick gold-plated copper wire needle soldered to the back of the brass plate of the diaphragm. The authors suggested that the probe allows the detection of sounds within a radius of 30–100 cm in the soil.

Recently, the set-up of choice has been to combine an audio recorder such as a Zoom H4N Pro or F6 or MixPre with an aluminium (Al) probe, a MicBooster pre-amp (increasing the gain by +20 dB), an impedance filter to reduce the unit noise (thereby improving the signal:noise ratio) and a contact microphone—also known as a piezoelectric mic [12]. When piezoelectric ceramics, such as lead zirconate titanate (PZT), are mechanically deformed, their crystal structure, which lacks a centre of symmetry, experiences a displacement of ions [59]. In their unstressed state, the centres of positive and negative charges coincide, resulting in no net dipole moment within the unit cells. The deformation caused by mechanical pressure (e.g. from vibrations generated by organisms) separates the positive and negative charges within the material, generating local electric dipoles [59]. As the dipoles align with the applied mechanical stress, a macroscopic polarization occurs, resulting in an electric field and surface charges on the ceramic. These surface charges can be collected using electrodes, producing an electrical charge proportional to the applied stress [59]. The piezoelectric effect, therefore, allows for the conversion of mechanical energy (e.g. acoustic wave) into electrical energy. Aluminium probes are considered optimal due to their density and sound transmission properties. For instance, lead (Pb) transmits sound at approximately 1210 m s^−1^, copper (Cu) at 4600 m s^−1^, steel or iron (Fe) at 5940 m s^−1^ and aluminium at 6420 m s^−1^ [60,61]. Moreover, pilot research has shown aluminium tri-pegs to outperform steel pegs [12]. Researchers should trial new materials, geometries, recorders and sensors to further improve the field. This might include a network of interconnected probes or sensors integrated with other field ecology systems (e.g. environmental data loggers). Improving sensor (mic) sensitivity and designing sensors specifically for different soil environments/properties (see Environment variables) would bring immense value.

**Figure 4.**
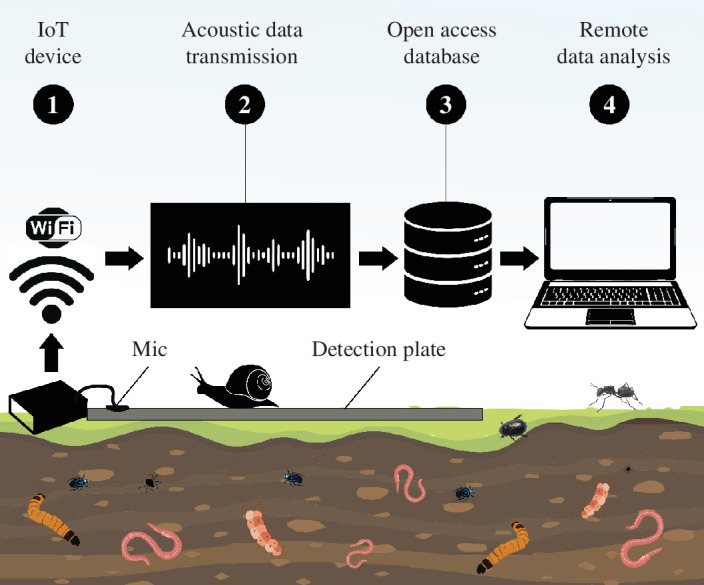
Detection plate innovation is currently being developed for surface-moving invertebrates. This could be combined with an Internet of Things (IoT) module to provide remote, real-time data transmission (see the IoT section for more information).

A novel tool currently being developed is a detection plate for surface-moving invertebrates (figure 4). This plate is aluminium, with sensors attached. The plate is laid out on the soil surface and is designed to detect organisms such as ants, snails, millipedes and spiders that move across the soil surface. Each group has unique morphological and behavioural phenotypes (e.g. size, weight, number of limbs, body texture and speed of movement) that create distinct acoustic signatures (figure 3).

### (c) Environmental and edaphic influences on soil acoustics

The acoustic properties of soils will change with factors that influence their structural and mechanical attributes, including texture, porosity, bulk density, moisture content and organic matter levels. Therefore, different soil types, climate, weather, land use and management (e.g. which influence organic matter levels, compaction by farm machinery or livestock, erosion and loss of topsoils) can all impact sound propagation in soils in various ways. Oelze *et al*. [62] examined the speed and attenuation of sound with varying soil types, moisture content and compaction. Using a hydrophone apparatus, they found attenuation coefficients over frequencies of 2–6 kHz ranging from 0.12 to 0.96 dB cm^−1^ kHz^−1^. They found sound attenuation correlated with increasing soil water content and compaction (i.e. bulk density), such that lower sound attenuation was generally observed in loose dry samples. In their system, very high attenuation (acoustically lossy) responses reduced the capacity for reliable signal detection above 6 kHz. They observed acoustic velocities of 86–260 m s^−1^ (compared with 343 m s^−1^ in air at 20°C) and decreasing acoustic velocity with increasing water content and total porosity. They also reported an acoustic detection range (in the context of detecting buried objects) of 40 cm below the soil surface. Lu *et al*. [63] studied the effects of compaction on soil acoustic velocity by subjecting soils to different levels of compression, designed to simulate compaction induced by agricultural machinery. They found acoustic velocity increased with compaction, reaching 400–1200 m s^−1^ in severely compacted soils. Lu and Sabatier [64] monitored temporal variation in acoustic velocities *in situ* in a trench containing a soil–sand mixture, with recordings in the range of 150–450 m s^−1^. Remarkably, they found that the speed of sound had an almost identical trend to the matric water potential (measured suction pressure of water bound to the soil matrix), i.e. sound travelled more freely as soils dried out. Suravi *et al*. [65] studied the influence of organic matter on acoustical properties, where organic matter introduces additional pore space and moisture-holding capacity to soils. When high- and low-organic content soils were compacted to the same density (1.3 g cm^−3^) then higher absorption coefficients (a component of sound attenuation) were observed in lower organic content soils. However, when the same soils were packed at constant pneumatic pressure (allowing settling at different densities), then higher absorption coefficients (across most of the frequency spectrum up to 1400 Hz) were observed in the high-organic content soils. Importantly, early research by Nyborg and Rudnick [66] suggests that wet soils can have markedly different attenuation properties if air is present in the pore spaces. Saturated, air-free soils had exceptionally low attenuation coefficients, while saturated soils prepared in the presence of air had much larger sound attenuation. Further research on the sound propagation potential of different soil properties should be a priority.

### (d) Data democratization

Data democratization in soil ecoacoustics will ensure that valuable ecological data are accessible and usable by a broad audience, including researchers, policymakers and the general public. Citizen science initiatives can empower individuals to actively participate in data collection and analysis [67]. By involving volunteers in soil ecoacoustics monitoring, the spatial and temporal coverage of data collection efforts can be expanded. This participatory approach can enrich the dataset and foster public engagement in soil conservation/restoration and environmental stewardship. The database mentioned above should be an open-source platform. By making software and datasets freely available, we can enable widespread access to ecoacoustics analysis tools. This transparency can promote collaboration, innovation and the replication of scientific studies, thereby enhancing the reliability and impact of ecoacoustics research. Providing comprehensive training resources also ensures diverse users can effectively contribute to and utilize soil ecoacoustics data. Online courses, workshops and tutorials (capacity building) can help individuals develop the necessary skills to engage in data collection, analysis and interpretation.

### (e) Internet of Things

The Internet of Things (IoT) is a network of physical devices embedded with sensors, software and other technologies to connect and exchange data with other devices and systems over the internet [68]. This connectivity allows real-time monitoring, data collection and automated decision-making processes across various industries [68]. IoT applications have been used in animal ecology and forest management studies; they are also increasingly used in the monitoring of agroecosystems. For instance, Wild *et al*. [69] found that Wi-Fi and smart-embedded software increased the retrieval efficiency of biologging data (collection of ultra-fine-scale animal movement data) by orders of magnitude compared with other available systems. Moreover, Almeida *et al*. [70] developed EdgeFireSmoke++, a novel lightweight algorithm for real-time forest fire detection and visualization using an IoT-human-machine interface.

Integrating soil ecoacoustics with IoT would involve connecting acoustic sensors to an IoT network. This could facilitate real-time data to be transmitted to cloud-based platforms for analysis, enabling more efficient soil health monitoring. This integration could lead to improved land management practices, early detection of soil health issues, more rapid/real-time responses to soil health issues, efficacy of pest management strategies and a better understanding of soil biodiversity. Expanding soil ecoacoustics hardware and software will involve refining sensor capabilities, enhancing data processing algorithms and optimizing connectivity protocols to integrate soil ecoacoustics monitoring systems into IoT networks. By leveraging IoT principles, soil ecoacoustics technology could potentially be scaled up for widespread deployment across diverse environmental settings, enabling real-time monitoring, remote data access and adaptive management strategies for soil health and biodiversity conservation initiatives (figure 4). IoT-soil ecoacoustics integration opens novel future avenues for interdisciplinary research collaborations and innovative solutions to help address complex environmental challenges on a global scale. The potential challenges of IoT-soil ecoacoustics integration might include data transmission reliability in remote areas, ensuring sensor durability in harsh soil conditions and managing the large volumes of data generated for effective analysis. However, these challenges can be overcome with further research and development.

## 4. Conclusions

As recently highlighted by Sutherland *et al*. [23] soil ecoacoustics has vast potential to improve the management, conservation and restoration of soil ecosystems in a non-invasive, cost-effective and efficient manner. By leveraging the capabilities of acoustic technology, researchers can gain valuable insights into the complex and dynamic ecosystems above- and below-ground. This review highlights the potential applications of soil ecoacoustics across land uses in different typologies of ecosystems/landscapes, demonstrating its potential in detecting and monitoring soil biota, assessing restoration efforts and evaluating agricultural practices. The current state of soil ecoacoustics, while promising, still faces several challenges, particularly in standardizing methodologies, data curation and sharing, isolating biophonic signals from background technophonies and developing robust analytical tools tailored for soil environments. Addressing these challenges by developing optimized hardware, advanced analytical pipelines, coordinated collaborative efforts and innovative experimental approaches will be crucial for advancing the field. Creating an international open-access database for soil ecoacoustics data will also be instrumental in fostering collaboration, standardization and data democratization within the scientific community. Given the demonstrable potential of ecoacoustics to improve soil ecological monitoring, we encourage the pursuit of further research and development as a priority.

## Data Availability

This article has no additional data.
